# Studies of a Naturally Occurring Selenium-Induced Microcytic Anemia in the Przewalski’s Gazelle

**DOI:** 10.3390/ani14071114

**Published:** 2024-04-05

**Authors:** Yang Ran, Yuanfeng Li, Xiaoyun Shen

**Affiliations:** School of Life Science and Engineering, Southwest University of Science and Technology, Mianyang 621010, China; yangran@mails.swust.edu.cn (Y.R.); lyf3092712072024@126.com (Y.L.)

**Keywords:** Przewalski’s gazelle, microcytic anemia, selenium nutrition, copper deprivation, the Qinghai–Tibet Plateau

## Abstract

**Simple Summary:**

This study was designed to find the cause of microcytic anemia in the Przewalski’s gazelle by investigating the mineral contents in its habitat. We found that the cause of the disease was selenium deficiency in the habitat, and then, a cure for the disease was discovered. It is extremely beneficial to protect the rare species of the Przewalski’s gazelle.

**Abstract:**

Due to the fencing of the Przewalski’s gazelle (*Procapra przewalskii*), the microcytic anemia incidence rate continues to increase. The primary pathological symptoms include emaciation, anemia, pica, inappetence, and dyskinesia. To investigate the cause of microcytic anemia ailment in the Przewalski’s gazelle, the Upper Buha River Area with an excessive incidence was chosen as the experimental pasture, and the Bird Island Area without microcytic anemia disease was chosen as the control field. Then, the mineral contents in the soil, forage, blood, and liver, as well as the blood routine parameters and biochemical indexes were measured. The findings showed that the experimental pasture had much lower Se content in the soil and forage than the control field (*p* < 0.01), while the impacted pasture had significantly higher S content in the forage. The damaged gazelles had considerably lower Se and Cu contents and higher S content in the blood and liver than the healthy gazelles (*p* < 0.01). The presences of Hb, HCT, MCV, and MCH were significantly decreased compared to those in healthy gazelles (*p* < 0.01). The experimental group had a significantly lower level of GSH-Px activity in their serums compared to the control group (*p* < 0.01). In the treatment experiment, ten gazelles from the affected pasture were orally administered CuSO_4_, 6 g/animal once every 10 days for two consecutive times, and all gazelles were successfully cured. Therefore, it is possible that low Se content in the soil induced an increase in the absorption of S content by forage, leading to the deficiency of secondary Cu in the Przewalski’s gazelles, resulting in microcytic anemia.

## 1. Introduction

The Przewalski’s gazelle is an endangered ungulate and endemic species in the Qinghai–Tibet Plateau of China. It is a member of the genus *Procapra*, subfamily *Antelopelae*, family *Bovidae*, order *Artiodactyla*, and class *Mammalia* [1,2]. Historically, this species had widespread distribution in Inner Mongolia, Ningxia, Gansu, Qinghai, Xinjiang, Tibet, and other regions [3,4]. Since the last century, the distribution range of the Przewalski’s gazelle has gradually narrowed because of some factors such as the increase in human population, expansion of human activities, grassland degradation, pasture fencing, poaching, habitat fragmentation, and other reasons, resulting in a sharp decrease in its population. Currently, the habitat of the Przewalski’s gazelle is retained only in the Qinghai Lake Basin [5,6]. From 1986 to 1994, the population was fewer than 300 individuals [7]. In 2003, there were about 600 gazelles [8]. Additionally, from 2009 to 2012, the population increased to about 1300 individuals [9]. Monitoring reports from the Qinghai Lake National Nature Reserve showed that there were about 3200 gazelles in 2020. According to recent studies, the Hudong-Ketu area, the Yuanzhe area, and the Bird Island refuge are among the isolated distribution points where the Przewalski’s gazelle primarily dwells [10,11]. It was once the least abundant among the endemic mammals in China. Moreover, the Przewalski’s gazelle was categorized as a category I species under China’s Wild Animal Protection Law and as an endangered species on the International Union for Conservation of Nature’s (IUCN) Red List of Endangered Species.

Selenium (Se) is an essential trace element for animals and plays a variety of roles in the body. Its biological function is mainly reflected in Se protein. Se usually exists in the form of selenocysteine in the catalytic center of selenoproteins [12]. And it is involved in antioxidant activity, immune regulation, endocrine function, bone metabolism, iodine metabolism, and reproduction [13,14]. The known selenoproteins include glutathione peroxidase (GSH-Px), phospholipid hydroperoxide glutathione peroxidase (PHGPx), and selenoprotein P. The GSH-Px is one of the most important members of cellular redox homeostasis, catalyzing the reduction of harmful peroxides and free radicals by selenocysteine in the catalytic domain of reducing glutathione [15]. Furthermore, it is also involved in cellular immunity, humoral immunity, and other non-specific immune functions [16]. Se deficiency can affect the proliferation and differentiation of lymphocytes, reduce the secretion of lymphocytes, and seriously inhibit the activity of lymphocytes and phagocytes. Therefore, Se is closely related to the disease resistance of animals. Se deficiency in animals can lead to stunted growth, decreased fertility, emaciation, degeneration and necrosis of skeletal muscle, degeneration of cell membranes, protein damage, DNA mutation, and in the most severe cases, premature aging and death of individuals, and even population collapse and species extinction. Similarly, when animals consume Se-rich plants, it usually accumulates in the form of selenate or Se-methylselenocysteine, resulting in acute selenosis and eventual death of livestock [17]. Long-term survival in a Se-rich environment can result in chronic selenosis in the Przewalski’s gazelle, which is marked by anemia, impaired antioxidant function, poor immunological function, and aberrant phenylalanine metabolism [18].

Copper (Cu) is an essential micronutrient for both animals and humans [19,20]. It plays a crucial role in cell transporters. Moreover, Cu serves as a cofactor for numerous crucial enzymes [21]. Therefore, it is vital for the normal functioning of biological metabolic processes, including hemoglobin synthesis, iron (Fe) oxidation, neurotransmitter synthesis, and antioxidant processes [22,23]. The Cu deprivation encompasses induced or direct types in animals. In the presence of sulfur (S), molybdenum (Mo) initially forms thiomolybdates with S in the rumen. Additionally, it forms a Cu-thiomolybdate complex with Cu in feed, resulting in inducing deprivation and reducing the absorption of Cu by organisms [24,25]. Simultaneously, Zn and Fe, along with higher protein content, also impact the absorption of Cu by animals [26,27,28]. The Cu deficiency in animals can result in anorexia, diarrhea, pica, emaciation, dyskinesia, and even death [29]. An overabundance of Cu can result in Cu poisoning. Acute and chronic Cu poisoning in ruminants are two different types of Cu poisoning. Rare in the wild, acute Cu poisoning is brought on by ingesting significant amounts of Cu compounds by accident. The long-term consumption of high Cu concentrations, which is more prevalent in nature, results in chronic Cu poisoning. Animals that consume large amounts of Cu over time accumulate the metal in their livers without exhibiting any symptoms. The accumulation of Cu can cause hemolysis, which can result in severe jaundice, hemoglobinuria, liver and kidney damage, and rapid mortality when it is beyond the liver’s tolerance zone [30]. In recent years, the microcytic anemia incidence rate of the Przewalski’s gazelle continues to increase in the Upper Buha River Area due to the fenced grassland. The main characteristics are emaciation, anemia, pica, inappetence, and dyskinesia. They are similar to the symptoms of Cu deficiency diseases. Red blood cells in microcytic anemia can divide, but because of poor heme synthesis or Fe binding, they typically cannot grow to their normal size. Lead poisoning, Fe insufficiency, and vitamin B6 inadequacy are the main causes of this kind of microcytic anemia [31].

The objective of this research was to investigate the mineral contents of the soil, forage, and animal tissues and to determine the physiological values and biochemical parameters, so as to elucidate the cause of microcytic anemia in the Przewalski’s gazelle and identify methods for its treatment.

## 2. Materials and Methods

### 2.1. Study Region

The study regions are located in the Upper Buha River Area and the Bird Island Area within the Qinghai Lake Basin. The region exhibits an average altitude ranging from 3036 to 3526 m and falls under the classification of a semi-arid alpine climate. One of the most striking features of the Qinghai Lake Basin is its diurnal temperature variation. Temperatures can fluctuate widely between day and night, creating a challenging environment for both plants and animals. The annual average temperature in the region ranges from 0.3 to 1.1 °C, with temperatures often dropping below freezing during the winter. Despite the harsh climate, the Qinghai Lake Basin experiences simultaneous rainfall and warmth in the same season. This unique weather pattern, combined with ample sunlight and a brief frost-free period, allows for the growth of a variety of plant species in the region. The predominant vegetation types are grassland and alpine meadow. These ecosystems support a diverse array of plant species, including *Puccinellia distans*, *Agropyron cristatum*, *Achnatherum splendens*, *Orinus kokonorica*, *Stellera chamaejasme*, *Artemisia desertorum*, and *Iris lacteal* [32].

### 2.2. Design Experiments

Experimental pasture: The Upper Buha River Area, exhibiting a high incidence of microcytic anemia in the Przewalski’s gazelles, served as the experimental pasture. The Bird Island Area without microcytic anemia was designated as the control pasture.

Experimental animals: In the Upper Buha River Area, ten male Przewalski’s gazelles, aged one year, were selected as the experimental subjects due to their microcytic anemia diseases. Ten male Przewalski’s gazelles that were one year old and in clinical good health were also chosen from the Bird Island Area to serve as the control group.

Clinical investigations: The Przewalski’s gazelles with microcytic anemia disorder activities on the pasture were observed directly to record clinical signs.

### 2.3. Sample Collection

Soil samples: An amount of 500 g of surface soil samples were taken at intervals of 200 m, measuring 1 m by 1 m and taken at depths of 0 to 20 cm. Ten soil samples were taken from the pasture under control and the pasture under experimentation.

Forage samples: A total of ten mixed samples, each weighing 500 g, were collected from the experimental and control pastures. The distribution of soil samples and mixed forage samples was the same.

Capture animals alive: In July or August, about 17–20 o’clock, the researchers concealed themselves 10–15 m away from the Przewalski’s gazelles and used a tranquilizer pistol (model: L50, Qinghai, China) to catch the animals alive. Anesthetic ketamine hydrochloride (approval number H35020148) was utilized. After injecting the gazelles with the anesthesia five times in three minutes at a dose of thirty milligrams each, samples of their liver and blood were taken. Then, they were released in situ. About twenty minutes later, the creatures were once again normal.

Blood samples: Ten mL of blood was collected from the jugular vein of each experimental gazelle using vacuum blood collection tubes without additives and containing dipotassium EDTA-K2, respectively. All collected bloods were transferred to the lab for analysis in less than four hours after being maintained and refrigerated at 4–8 °C.

Liver samples: One day before the procedure, the gazelles’ platelets and blood coagulation times were assessed, and the surgical puncture sites were found using ultrasound technology. On the day of the procedure, the gazelles were anesthetized with 0.09 g of codeine and 0.12 g of sodium phenobarbital, respectively, and 10 mg of vitamin K was given intramuscularly. The surgeon attached a rubber tube and hepatic puncture needle to a 20 mL syringe, took 3–5 mL of sterile saline, and then removed the gas from the syringe. In the beginning, a cone was used to make a hole in the animal’s skin, and a hepatic puncture needle was used to make a 0.5–1 cm puncture in the animal’s upper rib cage. To prevent the needle from becoming clogged, 0.5–1 mL of sterile saline was then administered into the liver puncture needle cavity to remove any remaining animal tissues. To establish and sustain negative pressure in the needle, it was drawn into the syringe’s 5–6 mL range. The liver tissue had already been quickly pierced vertically to a depth of around 4–6 cm when the gazelle held its breath. Following the removal of the liver puncture needle, the puncture site was compressed for a few minutes using sterile gauze and then secured with adhesive tape, compressed with a sandbag, and ultimately tightened with an abdominal belt. In order to fix it, the liver tissue from the liver puncture needle was put into a specimen bottle.

### 2.4. Sample Processing

The samples of soils and forages were air dried at 20 to 25 °C, then crushed and sifted through a 0.175 mm fine screen before being placed in bags for the subsequent test. An amount of 0.5 g of the material was placed into the digestive tube for the test, and 1 mL of hydrogen peroxide (H_2_O_2_) and 6 mL of nitric acid (HNO_3_) were added. After shaking the samples, they were left to stand for ten minutes. After dissolving the solution in accordance with the microwave digestion process, it was transferred to a 100 mL volumetric container and diluted to the appropriate level. And the marks were made right away.

The animal blood collected using vacuum blood collection tube without additives was centrifuged (3000 rpm/min for 10 min) to obtain serum. Whole blood samples and liver tissues were dried in air within 20–25 °C to constant weight, and then, they were put into a mixture (5:2:5) of HNO_3_, perchloric acid (HClO_4_), hydrofluoric acid (HF) and dissolved by microwave heating.

### 2.5. Sample Analysis

The Inductively Coupled Plasma Atomic Emission Spectroscopy (ICP-AES) (HK9600, Huaketiancheng Co., Ltd., Beijing, China) was used to assess the mineral contents of the soil, fodder, blood, and liver samples, including manganese (Mn), zinc (Zn), Fe, cobalt (Co), Cu, phosphorus (P), Mo, Se, and S. Laboratory quality assurance and quality control (QA/QC) procedures were carried out utilizing standard reference material (GSS-23) for soil samples and a spike-in experiment for blood and liver samples [33]. The validation of spike-in recovery of these mineral elements ranged from 85 to 107% for different concentration ranges. The specific recovery of each mineral element was as follows: Mn (97–103%), Zn (95–105%), Fe (96–107%), Co (90–102%), Cu (94–106%), P (92–101%), Mo (93–105%), Se (85–96%). The method detection limits (MDLs) were 0.002–3.000 μg·g^−1^ for the selected elements, and the relative standard deviation (RSD) of duplicate samples was between 3 and 35%.

The blood routine parameters, such as hemoglobin (Hb), white blood cell count (WBC), red blood cell count (RBC), hematocrit value (HCT), mean corpuscular volume (MCV), and mean corpuscular hemoglobin (MCH), were measured using the Automatic Blood Cell Analyzer (Sysmex Poch-100i Veterinary, Sysmex Corporation, Shanghai, China).

The Automatic Biochemical Analyzer (mindraybs-420, Mindray Biotechnology Co., Ltd., Shanghai, China) was used to assess biochemical parameters, such as GSH-Px, lactate dehydrogenase (LDH), aspartate aminotransferase (AST), total protein (TP), γ-glutamyl transferase (γ-GGT), alkaline phosphatase (AKP), creatinine (CR), and malondialdehyde (MDA).

### 2.6. Treatment Trial

Ten gazelles (1 year old, male) with microcytic anemia disease were selected and orally administered CuSO_4_ at a dosage of 6 g/animal once every 10 days for two consecutive treatments. Before and after treatment, their clinical symptoms were examined, and blood parameters and antioxidant indices were analyzed separately to determine the success of the treatment.

### 2.7. Data Analysis

SPSS software (SPSS, version 23.0, Inc., Chicago, IL, USA) was used to analyze the data. Data were analyzed by using independent samples of *t*-test to evaluate the significant differences among treatments and presented as means ± SD. Data in the treatment trial were compared overall by ANOVA and then compared between the after-treatment group and control group by Mann–Whitney *U* test to verify the significance of the differences.

## 3. Results

### 3.1. Clinical Investigations

As presented in Table 1, the main symptoms of the affected Przewalski’s gazelles were emaciation, anemia, pica, inappetence, and dyskinesia. For the majority of the time, there was no significant shift in body temperature, although occasionally, there was a little rise.

### 3.2. Content Analysis of Mineral Elements

In the afflicted region, the soil and mixed feed Se content was much lower than in the control area (*p* < 0.01). Furthermore, there was a significant difference (*p* < 0.01) in the S content between the mixed forage in the incidence region and the control area. There was no discernible variation seen in the remaining components (Table 2).

Compared to the normal gazelles, the afflicted gazelles’ blood and liver had far lower Se and Cu concentrations and higher S content (*p* < 0.01). There was no discernible variation found in the remaining components (Table 3).

### 3.3. Physiological and Biochemical Parameters Analysis

There was a significant difference in the levels of Hb, HCT, MCV, and MCH compared to the control group (*p* < 0.01). No significant difference was observed in other indicators. The aforementioned parameters indicated the presence of microcytic anemia ailment in the diseased Przewalski’s gazelles (Table 4).

Both the level of MDA and the activity of GSH-Px in the serum of the sick gazelles were substantially higher and lower (*p* < 0.01), respectively, than those in the control group (Table 5).

### 3.4. Treatment Experiment

**Figure 1:**
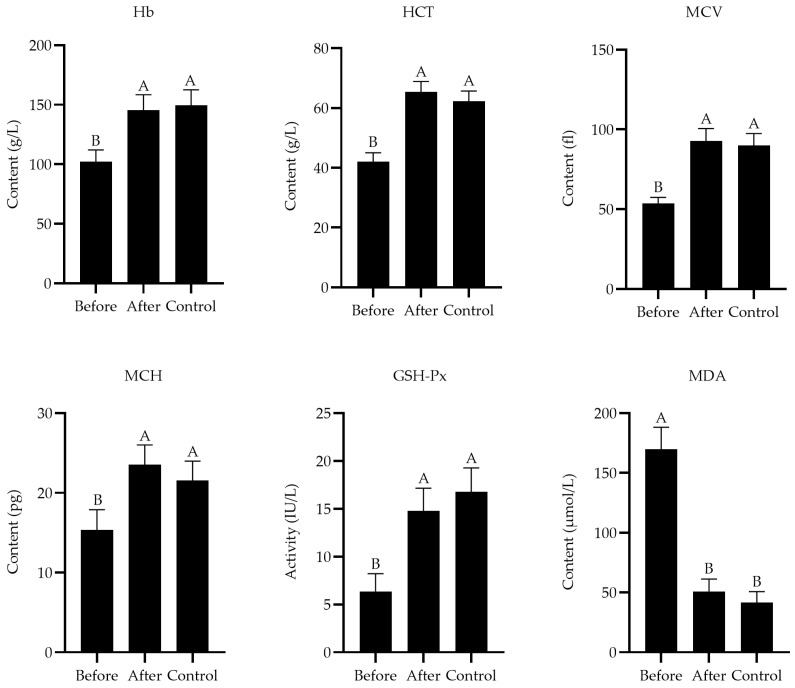
Effect of CuSO_4_ supplement on blood parameters and serum antioxidant indexes in Przewalski’s gazelles. Before = before the treatment experiment; After = after the treatment experiment; Control = control group. Different capital letters of superscript indicate very significant difference (*p* < 0.01), and the same letters indicate no significant difference.

After receiving treatment, the levels of Hb (F_2,27_ = 4.57, *p* = 0.004), HCT (F_2,27_ = 3.94, *p* = 0.002), MCV (F_2,27_ = 5.31, *p* = 0.003), and MCH (F_2,27_ = 4.29, *p* = 0.005) were elevated, the activity of GSH-Px (F_2,27_ = 2.93, *p* = 0.001) was increased, and the level of MDA (F_2,27_ = 5.64, *p* = 0.001) was decreased in the diseased Przewalski’s gazelles (Figure 1). There were no significant differences in these indicators between the after group and control group (*p* > 0.05). All Przewalski’s gazelles were successfully cured in the experimental intervention, and no other adverse reactions were observed.

## 4. Discussion

### 4.1. Mineral Contents in the Soil and Forage

The Qinghai–Tibet Plateau, often referred to as the “Roof of the World”, is a vast and diverse ecosystem that spans across multiple provinces in China and parts of Tibet. It is home to a unique array of flora and fauna, many of which are endemic to the region. The alpine meadows found in this plateau are particularly rich in biodiversity, harboring a significant gene pool of wildlife and plants. One of the key features of this region is the Buha River, which flows through the plateau and is the largest river in the Qinghai Lake area. The upper reaches of the Buha River are especially important as they provide a primary natural habitat for the Przewalski’s gazelle, a species that is well adapted to the harsh alpine environment of the plateau. Mineral elements are essential for the health and well-being of the wildlife and livestock that inhabit the Qinghai–Tibet Plateau. These elements play crucial roles in various biological processes, including metabolism, growth, and reproduction. In the case of the Przewalski’s gazelle, ensuring an adequate supply of essential minerals, such as Cu, is crucial for maintaining their health and reproductive success.

In this investigation, the Upper Buha River Area’s soil and mixed feed had a considerably lower Se concentration than the Bird Island Area’s (*p* < 0.01). And compared to the Bird Island Area, the mixed forage in the Upper Buha River Area had a considerably greater S content (*p* < 0.01). Se concentration in the soil and fodder below 0.1 μg·g^−1^ DM is generally considered inadequate. Moreover, Se content in the soil and forage below 0.040 μg·g^−1^ and 0.050 μg·g^−1^ DM, respectively, should be regarded as a significant indicator of Se insufficiency in ruminants [34]. This suggested that the lower Se concentration observed in the Upper Buha River Area’s soil and mixed feed could potentially lead to Se deficiency in the local ruminant population.

Alpine meadows are unique ecosystems found in high mountain regions, characterized by their cold climates and short growing seasons. These meadows are often covered with a variety of grasses, herbs, and shrubs, which form a dense network of interwoven root systems in the top layer of the soil. One of the key characteristics of alpine meadows is their low air permeability due to the intertwined root systems. This low permeability creates an anaerobic environment in the soil, which can have implications for the availability of certain nutrients, such as Se. In these anaerobic conditions, the oxidation of Se into selenate, a form that is readily taken up by plants, is hindered. This limitation can affect the concentration of Se in plants, potentially leading to Se deficiency in grazing animals that depend on these plants for nutrition [4]. In addition to Se, the absorption of other nutrients, such as S, can also be influenced by the competitive antagonistic relationship between selenates and sulfates. Both selenates and sulfates are absorbed through sulfate transporters in the root plasma membrane, and their absorption rates depend on the concentration ratio of selenates and sulfates in the soil [35]. Consequently, in Se-deficient soil environments, the limited availability of selenates can lead to an increase in the absorption of sulfates by plants.

The ability of feed to satisfy the Cu need of animals is mostly determined by the capacity for absorbing copper, rather than just the amount of Cu present in the feed. The reason for this is that when plants are high in S content, excess S combines with Cu and Mo in the rumen to form Cu-thiomolybdate complexes. It is not conducive to Cu absorption. In the intestines, thiomolybdate seals off Cu absorption sites and reduces Cu absorption. In the blood, Cu is not easily utilized by the tissues due to the formation of a relatively stable Cu–Mo–S–albumin complex. In the liver, thiomolybdate can directly strip Cu from metallothionein, and the stripped Cu can become a small molecule in the blood and bile. The metallothionein is then filled with Cu transferred from other proteins and then stripped again [24]. This cycle eventually leads to a decrease in Cu reserves in the animal, resulting in Cu deficiency. As a result, the absorption of Cu content by gazelles is reduced. Then, the secondary Cu content is deficient in the Przewalski’s gazelles. Generally, 7–8 μg·g^−1^ DM of Cu concentration in the feed suffices to meet ruminant needs. However, a higher Cu content is required in the mixed feed to meet the demands of the ruminants if they are exposed to an environment with excessive levels of Mo and S [3]. When animals are in a prolonged period of dry grass and receive insufficient amounts of vitamin C, vitamin E, and carotene from forages, along with inadequate intake of other nutrients, the signs of the illness will worsen [36]. This highlights the importance of providing animals with a balanced diet that meets their nutritional requirements, especially in environments where factors like high Mo and S levels can affect nutrient absorption and utilization.

### 4.2. Mineral Contents in the Blood and Liver

The mineral content of blood and liver are very important indicators, and they directly reflect the nutritional status of the animal [15]. And the hematological parameters serve as diagnostic indicators for assessing the degree of anemia in animals. The study found that the infected gazelles had considerably lower Se and Cu contents and higher S concentration than the healthy gazelles in the blood and liver (*p* < 0.01). Furthermore, compared to the healthy gazelles, the afflicted gazelles had considerably reduced the levels of Hb, HCT, MCV, and MCH (*p* < 0.01). The blood routine parameters revealed a substantial decrease in Hb values in gazelles, indicating that the Cu deprivation led to anemia in gazelles. Subsequently, the decreased levels of MCV and MCH indicated that the type of anemia in the Cu-deprived Przewalski’s gazelles was microcytic anemia disease.

Research in both animals and humans has demonstrated a significant correlation between Cu deprivation and anemia. Cu plays a crucial role in the body, particularly in the formation of red blood cells, as it is a cofactor for enzymes involved in hemoglobin synthesis and Fe metabolism [18]. In the case of Cu deficiency, these processes are impaired, leading to a reduction in Hb levels and disturbance in Fe metabolism. The former causes a decrease in hemoglobin content and a decrease in erythrocyte pressure volume, while the latter affects the size and morphology of erythrocytes, resulting in a smaller mean erythrocyte volume. This further results in a decrease in the average red cell hemoglobin volume.

Erythrocytes are particularly vulnerable to oxidative stress because their principal role is to transport oxygen. Erythrocytes in the circulatory system are constantly subjected to reactive oxygen species like H_2_O_2_ and superoxide. Thus, animals have developed a complicated antioxidant protection system that uses both enzymatic and non-enzymatic mechanisms to scavenge free radicals. The main components of the non-enzymatic system are vitamins, glutathione, Mn, Fe, Se, and Cu. The main components of the enzymatic system include SOD, CAT, GSH-Px, and other antioxidant enzymes [37]. The goal of the antioxidation effect in animals is to keep the body’s levels of oxidation and antioxidation in balance while removing excess free radicals. Once there is an imbalance in the synthesis and disposal of oxygen species, oxidative stress occurs, and erythrocytes are exposed to oxidative stress in the bloodstream, which can cause damage and trigger suicide death or erythrocyte apoptosis, resulting in anemia [38]. Se and Cu contents in the blood and liver significantly influence the activity of GSH-Px. Cu enhances the immune function of ruminants by augmenting the effect of neutrophils. Additionally, it influences antibody production, participates in the composition of immunoglobulins, and significantly increases the number of serum IgM and IgG, thereby enhancing the immune capacity of ruminants [39,40]. The main function of non-enzymatic systems in animals is to eliminate excess free radicals. The O_2_^-^ is effectively converted to H_2_O_2_ by SOD, then it is cleared by GSH-Px and CAT [41]. Furthermore, Cu is an important component of SOD [42]. Cu deficiency reduces the activity of Cu-Zn SOD and GSH-Px, stability of lipid peroxidation, and the amount of ceruloplasmin with antioxidant enzyme activity. They result in diminished antioxidant function, excessive free radicals, leading to pronounced oxidative stress and oxidative damage [43]. MDA, the most prevalent result of lipid peroxidation, directly represents the degree of lipid oxidative damage [44]. The higher the concentration of MDA, the more severe the oxidative damage to the body.

To address the microcytic anemia disease, it is crucial to ensure that the gazelles have access to a diet rich in Cu and that factors affecting its absorption, such as S, Mo, Zn, and Fe levels, are carefully managed. Monitoring the health and nutritional status of the gazelles and potentially supplementing their diet with Cu may be necessary to mitigate the risk of Cu deficiency-related diseases.

## 5. Conclusions

In view of the above findings, it can be concluded that low Se content in the soil led to increase in the absorption of S content by the forage. This, in turn, resulted in the deficiency of secondary Cu in the Przewalski’s gazelle, contributing to microcytic anemia in the gazelles. And the disease can be successfully treated with oral CuSO_4_.

## Figures and Tables

**Table 1:** Results of the clinical examination in the Przewalski’s gazelles with microcytic anemia disease.

Characteristic	Male (25) ^a^	Unpregnant (25) ^a^	Pregnant (25) ^a^	Antepartum (25) ^a^	Postpartum (25) ^a^
Occurrence of emaciation (%)	96	92	100	100	100
Occurrence of anemia (%)	100	100	100	96	100
Occurrence of pica (%)	100	100	100	100	100
Occurrence of inappetence (%)	92	96	100	100	100
Occurrence of dyskinesia (%)	20	24	52	64	72
Body temperature (°C)	37.59 ± 1.11	37.68 ± 1.13	37.55 ± 1.09	38.15 ± 1.14	37.34 ± 1.17
Heartbeat (beats/min)	56.92 ± 7.73	58.31 ± 9.56	59.46 ± 8.68	59.43 ± 9.18	58.86 ± 7.78
Breathing rate (breaths/min)	17.26 ± 2.61	17.28 ± 2.47	18.42 ± 2.41	19.31 ± 2.82	19.93 ± 2.28

^a^ Number of gazelle samples.

**Table 2:** The mineral contents of the soil and forage (μg/g).

Elements	Soil		Forage	
	Affected Areas	Healthy Areas	Affected Areas	Healthy Areas
Mn	111.15 ± 20.91	120.65 ± 25.65	64.42 ± 11.31	64.53 ± 12.46
Zn	33.41 ± 4.86	33.47 ± 5.01	7.39 ± 2.02	7.76 ± 1.87
Fe	6478.35 ± 712.47	6338.46 ± 697.38	536.79 ± 41.46	542.21 ± 38.43
Co	5.81 ± 1.15	6.77 ± 1.28	4.55 ± 1.11	4.15 ± 1.21
Cu	53.93 ± 18.81	52.41 ± 11.78	11.33 ± 2.19	11.14 ± 2.25
P	50.23 ± 6.05	51.19 ± 6.94	395.23 ± 55.11	398.05 ± 54.15
Mo	2.79 ± 0.48	2.7 ± 0.52	2.02 ± 0.37	2.01 ± 0.26
Se	0.020 ± 0.001 *	0.063 ± 0.003	0.028 ± 0.002 *	0.078 ± 0.003
S (%)	0.47 ± 0.14	0.44 ± 0.12	0.36 ± 0.02 *	0.21 ± 0.01

* When compared to the control, the difference was statistically significant.

**Table 3:** The mineral compositions of blood and liver in the Przewalski’s gazelles (μg/g).

Elements	Blood		Liver	
	Affected Gazelles	Healthy Gazelles	Affected Gazelles	Healthy Gazelles
Mn	0.79 ± 0.17	0.77 ± 0.16	4.34 ± 1.23	4.33 ± 1.26
Zn	3.33 ± 0.16	3.46 ± 0.11	50.23 ± 11.12	50.51 ± 10.78
Fe	353.76 ± 17.34	363.14 ± 17.57	441.25 ± 25.13	443.19 ± 24.27
Co	0.51 ± 0.11	0.49 ± 0.19	6.33 ± 1.21	6.42 ± 1.11
Cu	1.15 ± 0.12 *	1.67 ± 0.27	61.93 ± 8.42 *	97.85 ± 10.45
P	221.35 ± 19.95	223.25 ± 25.65	605.15 ± 35.15	589.95 ± 33.25
Mo	0.16 ± 0.03	0.17 ± 0.04	1.43 ± 0.11	1.62 ± 0.26
Se	0.012 ± 0.006 *	0.112 ± 0.062	0.117 ± 0.031 *	0.681 ± 0.068
S (%)	0.14 ± 0.03 *	0.09 ± 0.02	1.51 ± 0.05 *	0.96 ± 0.03

* When compared to the control, the difference was statistically significant (*p* < 0.01).

**Table 4:** Hematological values in the Przewalski’s gazelles.

Hematological Values	Affected Gazelles	Healthy Gazelles
Hb (g/L)	107.12 ± 10.76 *	149.59 ± 13.07
RBC (10^12^/L)	6.54 ± 0.22	6.58 ± 0.23
HCT (%)	39.05 ± 2.96 *	62.33 ± 3.34
MCV (fl)	56.67 ± 4.02 *	89.94 ± 7.53
MCH (pg)	15.52 ± 2.62 *	21.55 ± 2.44
MCHC (%)	22.09 ± 2.16	22.76 ± 2.05
WBC (10^9^/L)	8.99 ± 0.67	9.11 ± 0.64

* When compared to the control, the difference was statistically significant (*p* < 0.01).

**Table 5:** Biochemical values in the blood of the Przewalski’s gazelles.

Biochemical Values	Affected Gazelles	Healthy Gazelles
LDH (IU/L)	241.64 ± 49.51	237.09 ± 48.77
AST (IU/L)	24.67 ± 5.35	25.39 ± 4.95
γ-GGT (IU/L)	17.84 ± 3.45	17.81 ± 3.01
AKP (IU/L)	242.25 ± 12.35	247.95 ± 12.35
GSH-Px (IU/L)	4.43 ± 1.64 *	16.75 ± 2.54
MDA (μmol/L)	167.88 ± 18.69 *	41.54 ± 9.18
CR (µmol/L)	303.05 ± 32.31	295.45 ± 36.13
TP (mmol/L)	1.44 ± 0.22	1.49 ± 0.24

* When compared to the control, the difference was statistically significant (*p* < 0.01).

## Data Availability

The datasets generated and analyzed in the current study are available from the corresponding author upon reasonable request. The data are not publicly available due to privacy.
